# Supplementary material for: Exploring interactions between socioeconomic context and natural hazards on human population displacement
Source: Nat Commun. 2023 Dec 4;14:8004. doi: 10.1038/s41467-023-43809-8 (PMC10695951; doi:10.1038/s41467-023-43809-8)
Supplement: Supplementary file 1 — Supplementary Information [file 41467_2023_43809_MOESM1_ESM.pdf]

Supplementary Information

Shapley values: theory, properties, and code

Machine learning models are becoming so big and overparametrized that it is very difficult to understand or study what mechanisms they use for prediction. Many methods exist in the field of XAI under different learning paradigms, ranging from for *post-hoc interpretability* via advanced feature ranking techniques to *interpretability by design* of models via base visualization of the neural networks and neuron-integrated gradients on autoencoders. One of the methods used here is based on the Shapley Value sampling theory, which addresses the issue of subdividing the output of an arbitrary ML model among its input features in an optimal way. The Shapley values were originally introduced in the context of cooperative game theory to understand each player's contribution to the outcome of a game. However, they have found numerous applications in different fields, including ML.

Given a specific instance x, the objective is to decompose the prediction $f(x)$ among the multiple features x_i, eventually assigning Shapley values to each of them. Such scores can be regarded as a local explanation of the model output for this specific point. If we have $\mathscr{N} = \{1,\dots,n\}$ players (or covariates in the case of ML), then each non-empty subset $\mathscr{S} \subseteq \mathscr{N}$ forms a possible *coalition*. We can then consider the pair $(\mathscr{N}, v)$, where $v : 2^{\mathscr{N}} \to \mathbb{R}$ is the coalition function assigning an importance score to each coalition and such that $v(\emptyset) = 0$.

The Shapley value for any cooperative game $(\mathscr{N}, v)$ is then given by:

$$\phi_i^{Sh} = \frac{1}{|\Pi(\mathscr{N})|} \sum_{\pi \in \Pi(\mathscr{N})} \underbrace{[v(\mathscr{P}_i^{\pi} \cup \{i\}) - v(\mathscr{P}_i^{\pi})]}, \tag{1}$$

that is the average of all player's marginal contributions in permutation π. Therefore, a player's Shapley value is the player's average marginal contribution to the value of the predecessor set over every possible permutation of the player set.

The same concept can be transferred to the covariates of an ML model. However, the computation of the exact Shapley values requires an exponential number of characteristic function evaluations, resulting in exponential time complexity. This is prohibitive in most ML tasks where each evaluation can correspond to training a new model. For this reason, different methods to approximate the Shapley values have been proposed. Let us denote with $\widehat{\phi}_i^{Sh}$ the approximated Shapley value for the feature $i \in \mathscr{N}$. A linear time approximation based on the solution of the following optimization problem is:

$$w_{\mathscr{S}} = \frac{|\mathscr{N}| - 1}{\binom{|\mathscr{N}|}{|\mathscr{S}|} |\mathscr{S}| (|\mathscr{N}| - |\mathscr{S}|)} \tag{2}$$

$$\min_{\widehat{\phi}_0^{Sh},\dots,\widehat{\phi}_n^{Sh}} \sum_{\mathscr{S} \subseteq \mathscr{N}} w_{\mathscr{S}} \left(\widehat{\phi}_0^{Sh} + \sum_{i \in \mathscr{S}} \widehat{\phi}_i^{Sh} - v(\mathscr{S}) \right) \tag{3}$$

$$\text{s.t.} \quad \widehat{\phi}_0^{Sh} = v(\emptyset), \qquad \widehat{\phi}_0^{Sh} + \sum_{i \in \mathscr{N}} \widehat{\phi}_i^{Sh} = v(\mathscr{N}). \tag{4}$$

where $\mathscr{N} = \{1,\dots,n\}$ be the finite set of covariates, each non-empty subset $\mathscr{S} \subseteq \mathscr{N}$ is a coalition of covariates, and $\phi(\mathscr{N}, v) \in \mathbb{R}^{\mathscr{N}}$ is the *solution vector* to the cooperative game $(\mathscr{N}, v)$. The definition of weights in (2) and the objective function in (3) implies the evaluation of $v(\cdot)$ for 2^n coalitions. This complex problem has been addressed by subsampling the coalitions. The above method is implemented in the SHAP package, which we used to explain and interpret the NDP predicted by the RF and GBM models.

In Fig. 1, we show the average Shapley values for all test set random configurations, including only Asia (A), Africa (B), (C) North America, and (D) South America, respectively. Taking into account the reduced amount of data points, we used, in this case, random splitting with a ratio of 60/40 for the training and the test sets. Except for the model trained over Asia, which has $R^2 \sim 0.41$, the different RF models typically reach a lower R^2 of ~ 0.22 for Africa, ~ 0.18 for North America, and ~ 0.21 for South America. Notably, the same associations hold across continents, reinforcing the findings' generality. However, it is also interesting that the average importance (i.e., the ranking) can change significantly, suggesting specific features that characterize each continent. In particular, exposure factors, such as area for Asia and North America, elevation for Africa, and the fraction of cultivated land for South America, emerge as the most important predictors. Precipitation consistently remains among the most relevant predictors, with the second-highest score in three out of four configurations.

In Fig. 2, we show the average Shapley values for all test set random configurations, including only flood (A), storm (B), and landslide (C) events, respectively. We used a ratio of 60/40 for train and test sets. The flood model reaches a R^2 of ~ 0.31, the storm model of ~ 0.47 and the landslide model ~ 0.22. It is interesting to comment on the differences between the relations found by the three RF models. In particular, if we look only at storms (B), then vulnerability (i.e., AWI, Conflict fatalities) and exposure (i.e., area, %AgriLand) factors are more important than hazard properties in determining the amount of NDP. This

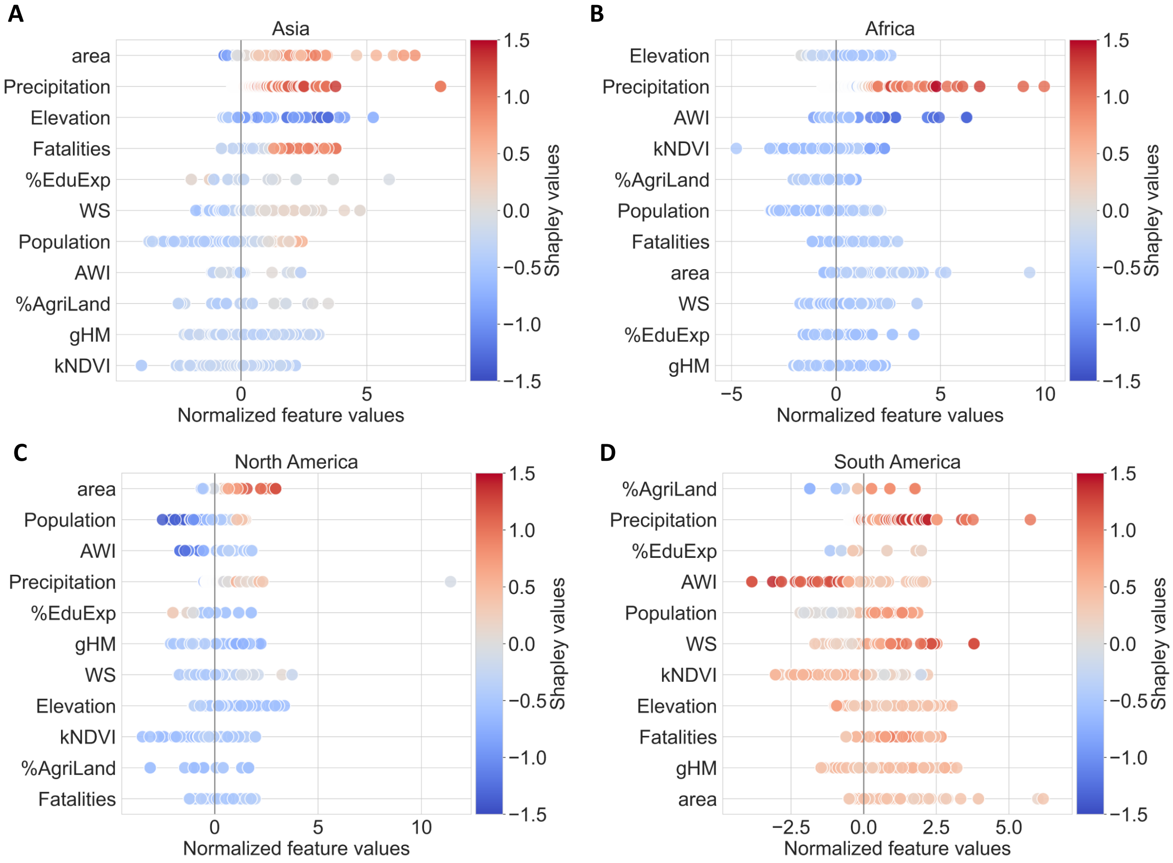

Figure 1. Average Shapley values considering: (A) only events occurred in Asia, (B) only events in Africa, (C) in North America, and (D) in South America.

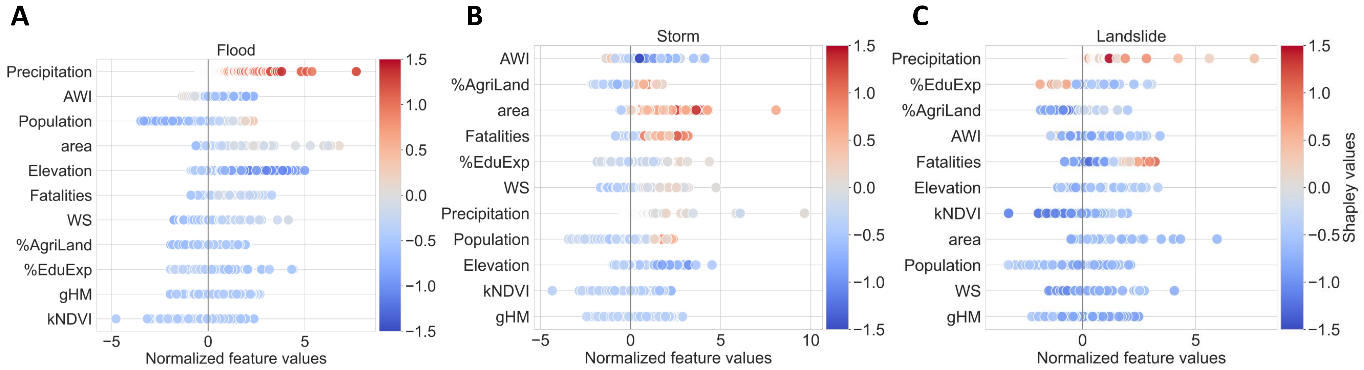

Figure 2. Average Shapley values obtained by disaggregating the type of hazard. Models trained only with: (A) flood events, (B) storm events, and (C) landslide events.

might be because, on average, storms are more destructive hazards than floods, and, thus, how many people will be forced to move depends mostly on how vulnerable and exposed the impacted area is rather than the specific meteorological characteristics of the event. Reasonably, WS becomes more relevant than precipitation for storm events, while both flood and landslide NDP are influenced more by precipitation. AWI is among the four most important variables for all three configurations, while %AgriLand is relevant only for storms and landslides but not flood events. This might be explained by the fact that storms and landslides are the hazards that cause greater damage to cultivated fields. As expected, elevation has an evident trend for floods, mainly in low-altitude regions that host river basins or can be affected by coastal flooding (Shapley values are negative for high-elevation values). It is interesting to observe that both area and population have a similar impact on storms and floods

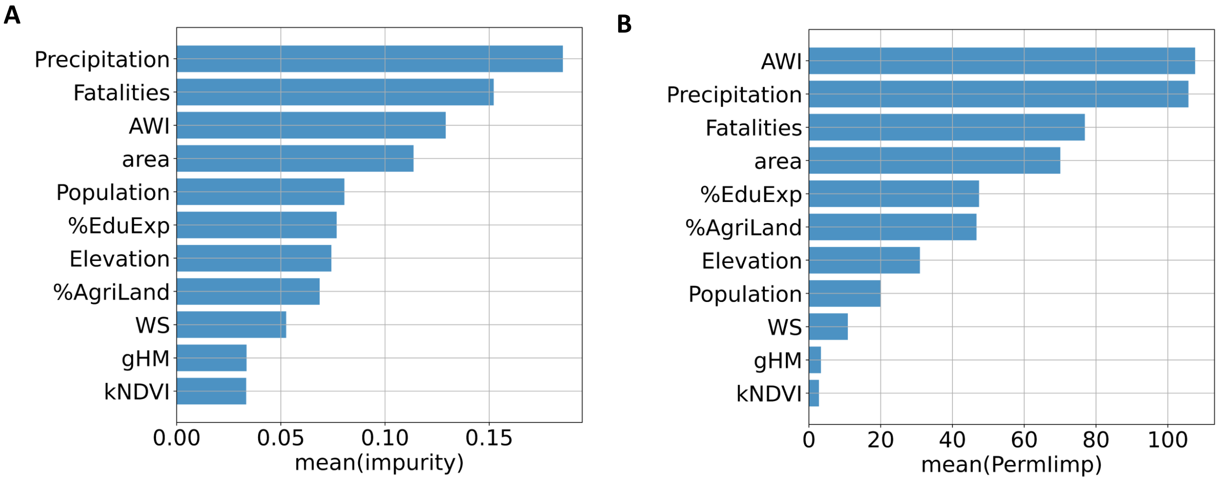

Figure 3. Feature importance of the RF models obtained with two different algorithms: (A) average impurity decrease, (B) random permutations of feature values.

but lower importance (and also a less clear trend) for landslides. Conflict fatalities play a comparable role across all three different hazards, as one might expect, assuming little or no correlation between the type of sudden-onset hazard and the deaths due to conflicts. Finally, the kNDVI has low importance scores for both storms and floods. Still, instead, it is relevant for landslide NDP, where there is a direct proportionality between NDP and kNDVI, suggesting that movements due to landslides are concentrated in agricultural or forest areas with medium-high kNDVI values.

In Fig. 3, the covariates are ranked using two different criteria, i.e. *impurity* and *permutation*. The former estimates the importance of each predictor based on the average decrease in the impurity of the leaves produced when that predictor is used to create a split in the tree. The final score is then obtained by averaging over all the trees in the RF. The latter ranking is instead produced by randomly permuting a given feature's values and observing the produced degradation in the model's output. Such permutations can eliminate (or reduce) the relation between the target and the covariate; thereby, the higher the change produced by a permutation in the output, the higher the importance of a covariate. Notice that even if the ordering and the quantitative scores differ in the two cases, the top four (and the last three) most important features are the same. As discussed in the main text, Precipitation is the main hazard factor, AWI is the most relevant covariate characterizing vulnerability, and area accounts for the exposure. This gives an overall view of the relative importance of the input features and confirms that results obtained with different XAI methods are consistent among them. However, unlike Shapley values, these two methods do not provide detailed information on the direction of the relationship between the covariates and target variables.

To extract additional insights and information on how the different covariates interact between them and influence together the RF model, we show the scatter plots of the Shapley values per event for other pairs of input variables. Since in the bootstrapping, the test set is randomly sampled with replacements, which means that the same points can be sampled again in different iterations, the Shapley values per event are computed as averages over all the values obtained for the same event by other RF models (i.e., in various iterations where a given event appeared). In Fig. 4, we further explore the hypothesis of differential vulnerability by studying the interaction of the Shapley values and the feature values for different combinations of covariates. In particular, we investigate the relationship between hazard (i.e., precipitation), land exposure (i.e., %AgriLand), and vulnerability (i.e., AWI and %EduExp). In Fig. 4A, we show the scatter plot of the Shapley precipitation values with the color scale given by the percentage of national education expenditures. Given a hazard with a fixed intensity, we observe that the number of resulting NDP is higher in areas with lower %EduExp, which are more vulnerable and have limited coping strategies to face disasters. In Fig. 4B, we show again the Shapley precipitation values with the color scale given by the percentage of agricultural land in a country. In similar precipitation levels, we observe that NDP tends to be higher in countries with a greater dependence on agriculture. This association may be linked to the vulnerability of livelihoods tied to agriculture, potentially leading to more frequent forced displacements than in urban or industrialized countries, although we recognize that agricultural dependence can also correlate with other factors, such as wealth and conflict. In Fig. 4C, we look at the interaction between human exposure (i.e., the number of people living in the affected area) and economic vulnerability (i.e., AWI). In this case, we find again evidence of differential vulnerability since events in highly populated regions mainly have low AWI values. Finally, in Fig. 4D, we analyze the Shapley values of conflict fatalities for two levels of AWI (i.e., above or below 650 USD). The interaction here is more complex. As expected and previously discussed, disasters in areas already affected by armed conflict lead to more displacement. It is not entirely clear at this stage whether low AWI values further amplify this effect. We note that

the choice of thresholds is primarily for illustrative purposes. However, the specific details of the interactions may indeed vary depending on the variables considered, and consequently, the most suitable threshold may also change. A quantitative study of such interactions could be performed in subsequent analyses. Additional plots are shown in Fig. 5.

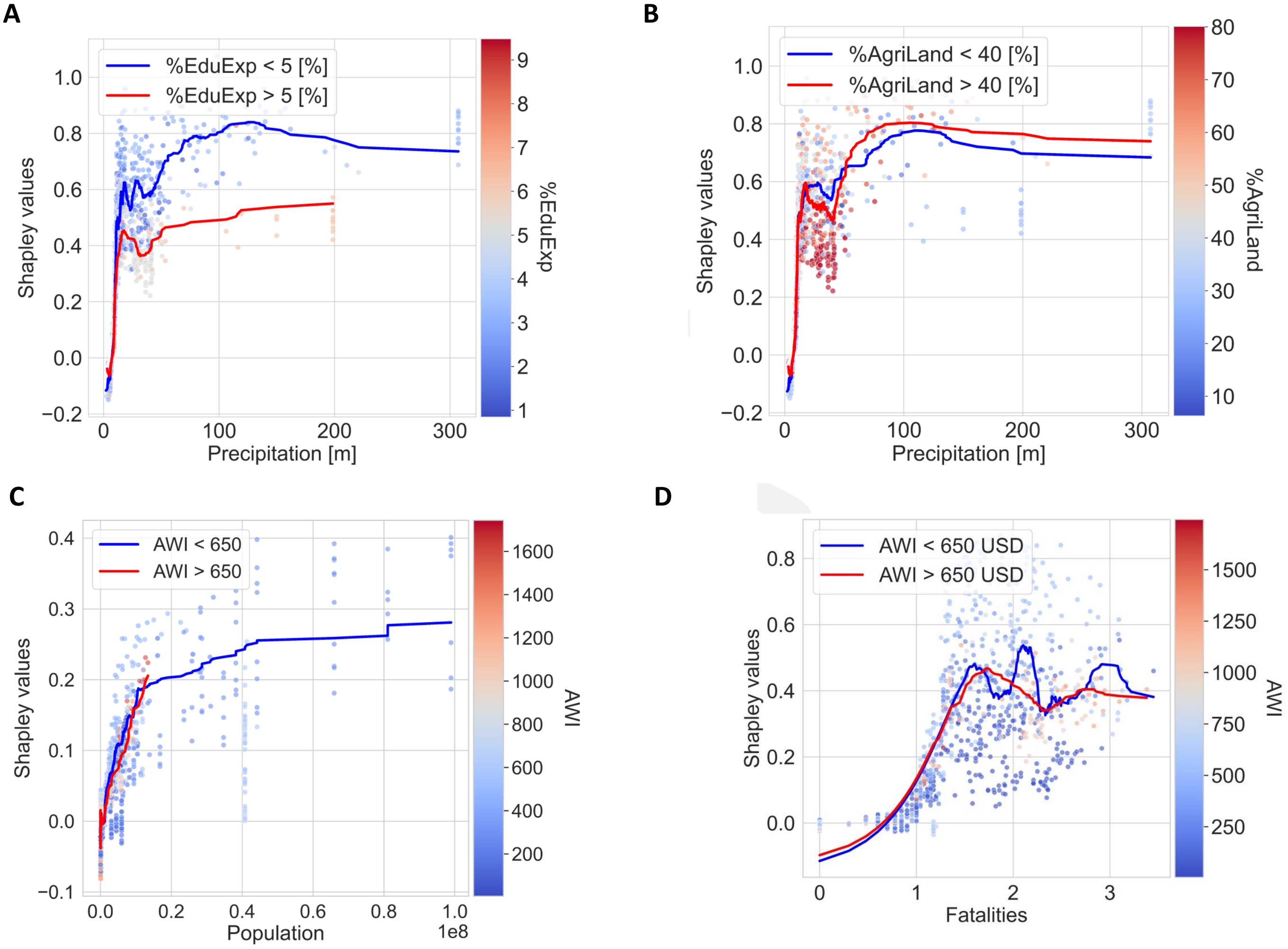

Figure 4. Scatter plots of Shapley values versus precipitation (A, B), Population (C), and Conflict fatalities (D). The color scale is given by the value of the percentage of education expenditures (A) and the percentage of agricultural land (B) or the value of the AWI (C, D). The blue and red curves are smoothed averages of the Shapley values for instances below or above a chosen threshold, varying depending on the variable considered.

Individual conditional expectations: theory, properties and code

Another way of extracting information from trained ML models for specific instances is given by the so-called Individual Conditional Expectations (ICE) plots. Each curve in the ICE plot represents the response obtained by varying the value of a specified covariate x_S conditioned on all the other ones x_C that are kept fixed, i.e., $\widehat{f}_i(x_S) = f(x_S, x_C \equiv x_C^i)$ being i the instance of interest or, equally, one of the curves in the ICE plot. In other words, each curve illustrates the conditional relationship between the covariate x_S and the model function f at fixed x_C. We compute the ICE plots for all the predictors and show them for 10 events randomly drawn from the test set (i.e., the different colored curves in Fig. 6, Fig. 7, and Fig. 8). Displaying more curves would make the plots harder to visualize without substantially changing the qualitative trends we find. To make the interpretation easier, we also re-scaled the curves by subtracting the average prediction per instance, i.e., $\widehat{y}_i = \widehat{f}_i(x_S) - \langle \widehat{f}_i(x_S) \rangle_{x_S^i}$ where $\langle f \rangle_x$ is the mean over the values of x_S, for the instance i. Results are shown in Fig. 6, Fig. 7, and Fig. 8. First, it is remarkable that we find not only the same relationships but also similar functional dependencies with a different XAI method. Compare, for instance, the ICE plots in Fig. 6A and Fig. 6C with the Shapley values in the main text. This is important as it gives us confidence that we might have uncovered the correct learned patterns from the trained *black-box* RF models. Then

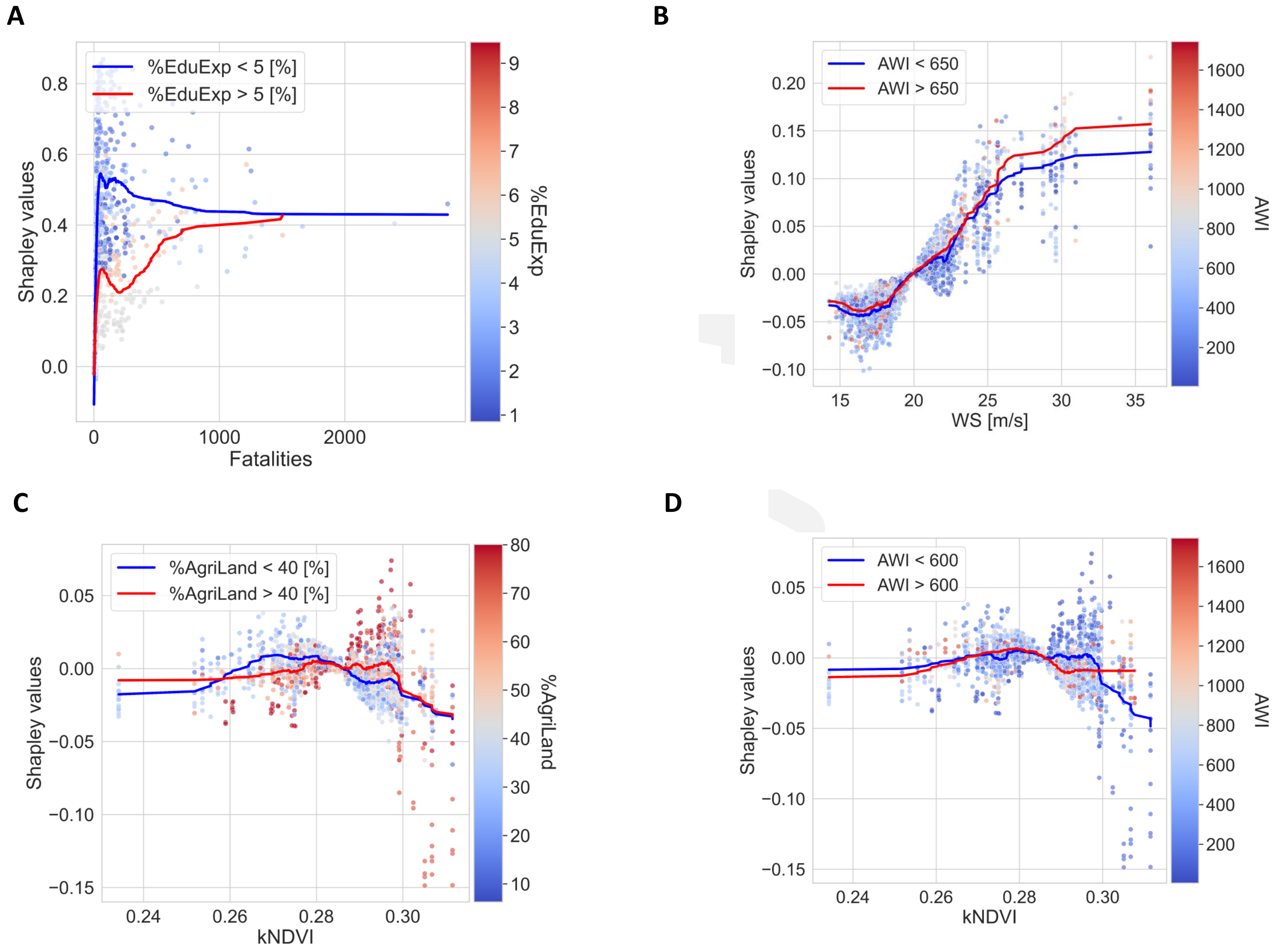

Figure 5. Scatter plots of Shapley values versus conflict fatalities (A), wind speed (B), and kNDVI (C, D). The color scale is given by the value of the percentage of education expenditures (A), the value of the AWI (B, D), and the percentage of agricultural land (C). The blue and red curves are smoothed averages of the Shapley values for instances below or above a chosen threshold, varying depending on the variable considered.

the span in the vertical axis (i.e., $\max(\log_{10}(NDP) - \langle\log_{10}(NDP)\rangle) - \min(\log_{10}(NDP) - \langle\log_{10}(NDP)\rangle))$ gives an estimate of the overall contribution of each predictor. Such scores agree with the ranking obtained with Shapley values, permutation importance, and impurity decrease. Finally, we find again highly interpretable non-linear dependencies between NDP and the identified drivers. It is also worth mentioning that the fact that the different curves in the ICE plots follow roughly the same trend (this is true, especially for the most important features, such as the area or Precipitation) means that there is limited interaction among the covariates, as we already discussed in other sections from different perspectives.

Causal Forest: theory, properties and code

To evaluate the causal effect of the variables in our problem, we rely on the EconML Python library and the Double Machine Learning (DML) model defined as follows:

$$\begin{aligned} Y &= \theta(X) \cdot T + g(X,W) + \varepsilon \qquad \mathbb{E}[\varepsilon|X,W] = 0 \\ T &= f(X,W) + \eta \qquad \mathbb{E}[\eta|X,W] = 0 \\ & \qquad \mathbb{E}[\varepsilon \cdot \eta|X,W] = 0 \end{aligned}$$

where $\theta(X)$ is the treatment effect we would like to estimate, Y is the outcome, T is the treatment or intervention applied, X is a set of observable characteristics, W is a set of variables that could have potentially influenced the choice of T, while also

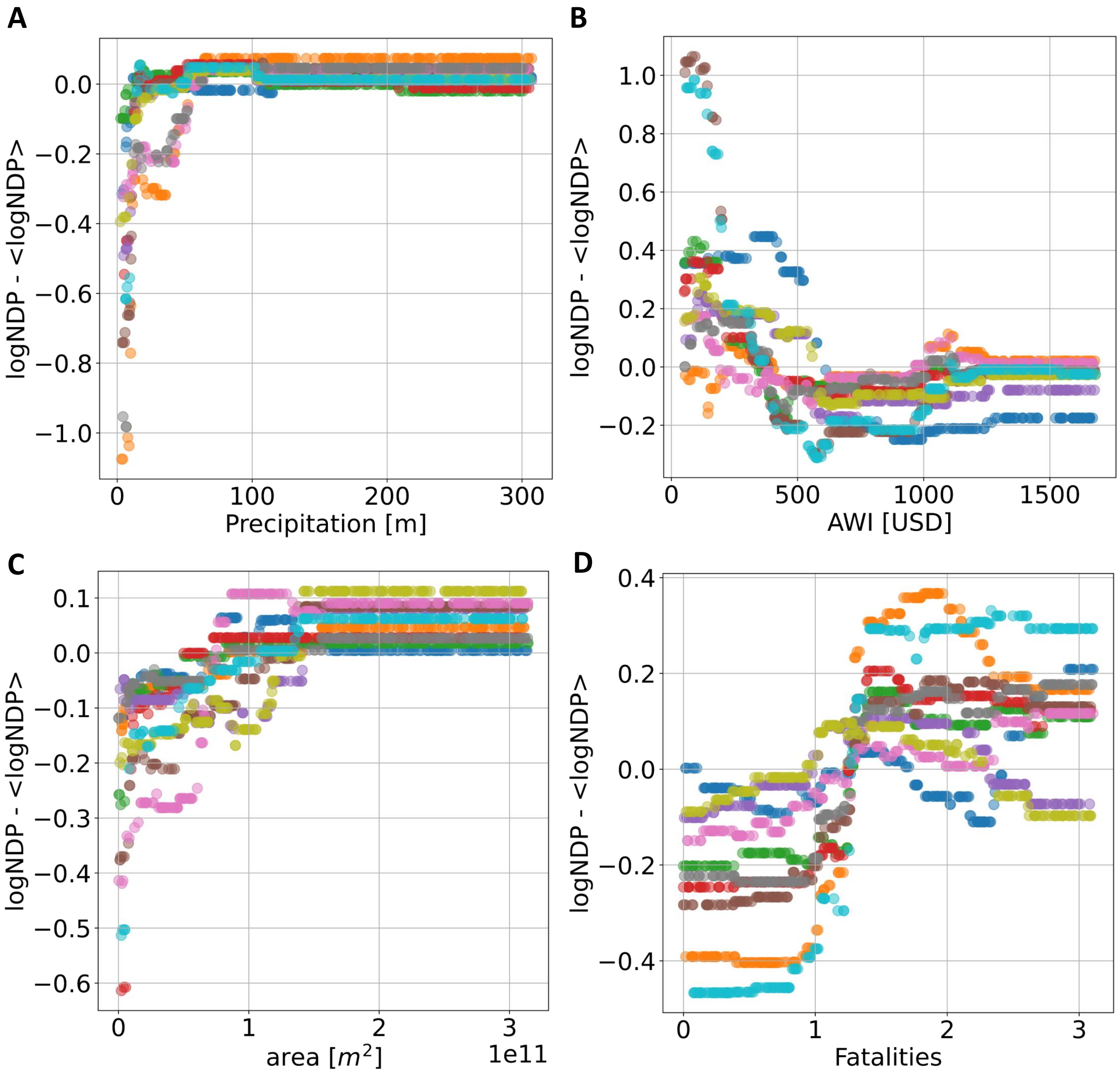

Figure 6. ICE plots of 10 randomly drawn instances in the test set for the following predictors: (A) Precipitation, (B) AWI, (C) area, and (D) Conflict fatalities. The x-axis is obtained by varying the values of the chosen predictor for a given data point, and the y-axis is the corresponding variation of the output, i.e., the (logarithm of) NDP. For visualization purposes, we subtract the mean NDP per instance. Each line (color) represents a different random instance.

directly affecting Y, and g and f are expectation functions defined as

$$g(X,W) = \mathbb{E}[Y|X,W]$$
$$f(X,W) = \mathbb{E}[T|X,W]$$

In our setting, to estimate the effect on a given variable, we consider that variable as the 'treatment' T and the rest of the variables as covariates, i.e., X following the previous notation. The causal effect can be defined as the extent by which Y is changed by a unit-level interventional change in T. The difference between the outcome obtained from the conditional mean in the presence of the treatment and the outcome obtained without the treatment (i.e., control group) determines the magnitude of the average treatment effect (ATE) or *causal effect*. To simplify the problem, we neglect the variables W in the light of the fact that the correlations among covariates (both Pearson and Spearman) are very low (less than 0.5 in all cases, except for *Population* and *area* which are understandably related to some degree), see Fig. 14. Consequently, we can reasonably assume the variables do not affect the 'treatment' variable (i.e., we can omit considering some variables as controls).

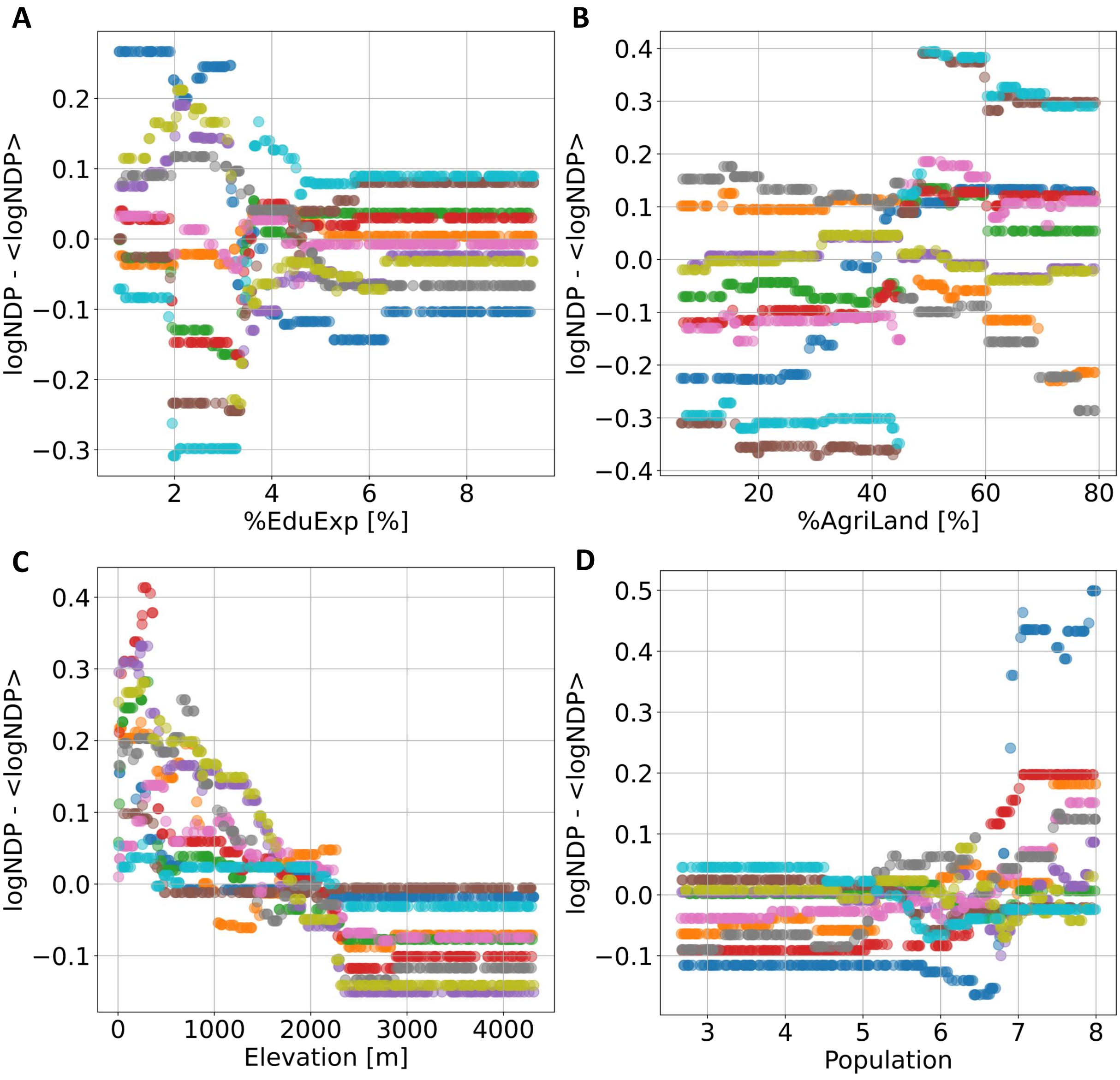

Figure 7. ICE plots of 10 randomly drawn instances in the test set for the following predictors: (A) %EduExp, (B) %AgriLand, (C) Elevation, and (D) Population. The x-axis is obtained by varying the values of the chosen predictor for a given data point, and the y-axis is the corresponding variation of the output, i.e., the (logarithm of) NDP. For visualization purposes, we subtract the mean NDP per instance. Each line (color) represents a different random instance.

The expectations g and f are estimated using a regression algorithm, in our case, a Random Forest. Then the following residuals are obtained as $\tilde{Y} = Y - g(X,W)$, $\tilde{T} = T - f(X,W)$, and $\theta(X)$ is estimated by solving the regression problem in the equation $\tilde{Y} = \theta(X) \cdot \tilde{T} + \varepsilon$. The final model was obtained using Causal Forest.

Causal Forests provide a method allowing one to estimate the heterogeneous causal effect of a treatment on individual samples or subjects, including confidence intervals on the estimations. They adapt Breiman's Random Forests non-parametric algorithm to the causal paradigm. In CF, forests of *causal trees* (rather than standard decision trees) are grown and aggregated. As the splitting criterion optimizes treatment effect heterogeneity, these causal trees estimate the causal effect on their leaves. Generalized Random Forests, which allow estimating conditional average partial effect and heterogeneous treatment effects with instrumental variables, provide in both cases valid confidence intervals. Table 1 summarizes the ATE for all the predictors with the associated confidence interval. It is worth mentioning that overall, the results in Table 1 are consistent with the Shapley values. Notice, for instance, that the precipitation treatment has a positive ATE, meaning that it increases NDP, as already found in multiple ways. On the other hand, the AWI treatment has a negative ATE and, thus, has the opposite effect on displacements.

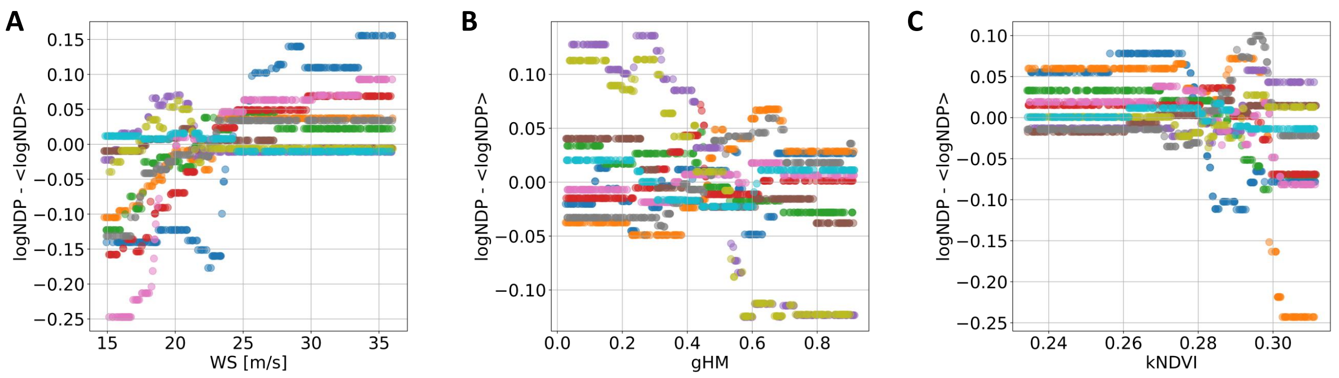

Figure 8. ICE plots of 10 randomly drawn instances in the test set for the following predictors: (A) WS, (B) gHM, and (C) kNDVI. The x-axis is obtained by varying the values of the chosen predictor for a given data point, and the y-axis is the corresponding variation of the output, i.e., the (logarithm of) NDP. For visualization purposes, we subtract the mean NDP per instance. Each line (color) represents a different random instance.

Table 1. Average treatment effect for all the covariates estimated using the causal forest algorithm and associated error bars.

**Covariate**	**Causal effect**	95% **confidence level**
Precipitation	0.25	[-0.15, 0.64]
AWI	-0.19	[-0.89, 0.50]
%AgriLand	0.16	[-0.39, 0.72]
Population	0.14	[-0.22, 0.50]
%EduExp	0.11	[-0.32, 0.54]
area	0.09	[-0.22, 0.41]
Elevation	-0.08	[-0.29, 0.14]
WS	0.07	[-0.08, 0.22]
Fatalities	0.05	[-0.17, 0.27]
kNDVI	-0.02	[-0.15, 0.10]
gHM	-0.003	[-0.32, 0.32]

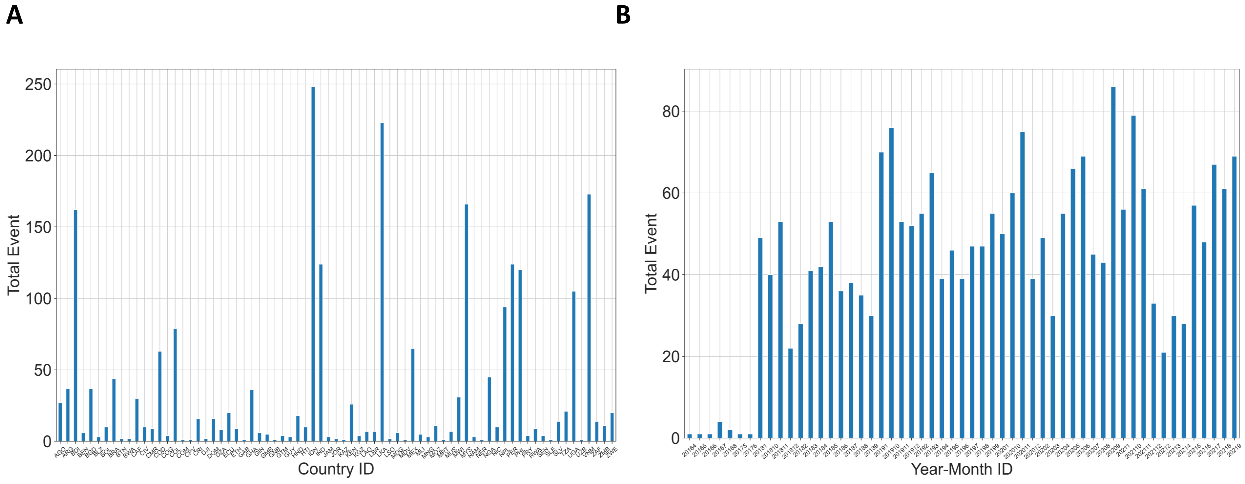

Figure 9. Cross-validation schemes. (A) Histogram of the events per country. (B) Histogram of the events per year-month pairs.

Cross-validation: spatial and temporal splits

We also considered different schemes for training and validating RF models to provide further evidence of the presented results. Specifically, instead of doing random splits with sizes of 70% and 30% for the training and testing sets, respectively, we explicitly took into account the temporal and spatial distribution of events in the data set. We studied how it affects models in terms of performance and the learned associations between NDP and the predictors. This procedure is helpful to strengthen the results further and check for possible confounding effects due to geographical or temporal characteristics in the collected data. However, it is worth remembering that all events in the dataset come from different areas and occur at other times, so random splits are already, to some extent, also temporal and spatial splits. In Fig. 9, we show the distribution of points for the spatial splits (A) and the temporal split (B). Note that there are significantly less data points for events occurring on 2016 given that IDMC implemented that year the methodology for monitoring disaster-related displacement.

Table 2. Performance of the RF models trained with temporal and spatial cross-validation.

Metric	RF (spatial)	RF (temporal)
R^2	0.10 ± 0.07	0.33 ± 0.02
RMSE	1.10 ± 0.06	0.96 ± 0.02
ME	-0.001 ± 1.1	-0.002 ± 0.93

In the first case, the data is grouped by country, while the second is by year and month. Since considering all possible combinations would be unfeasible in both cases, we randomly sampled the events using their spatial or temporal labels with a ratio of about $60/40$ percent for train and test. Metrics are reported in Table 2 and the Shapley values in Fig. 10. The R^2 obtained with temporal splittings is compatible with that obtained with random assignments. At the same time, the significant drop in the R^2 for the country-based splits can be explained by the fact that the records are not evenly distributed among countries, see Fig. 9. Moreover, the unique characteristics of each country may affect the generalization of the model trained on a specific set of countries. For instance, the model's ability to generalize may be higher among countries of the same continent (refer to earlier sections on continent-level experiments). Nonetheless, the top three features are the same for spatial and temporal cross-validation. They agree with those found with random splitting. Notably, even if the rankings show minor differences in the ordering, very similar relationships hold under the three different cross-validation settings: random, country-based, and based on year-month values.

Database: collection, sources and details

The target considered is the total number of NDP per event as registered by IDMC in the years $2016-2021$. The distribution of disaster events per continent is shown in Fig. 11. Notice that Asia has almost double the natural hazards concerning Africa,

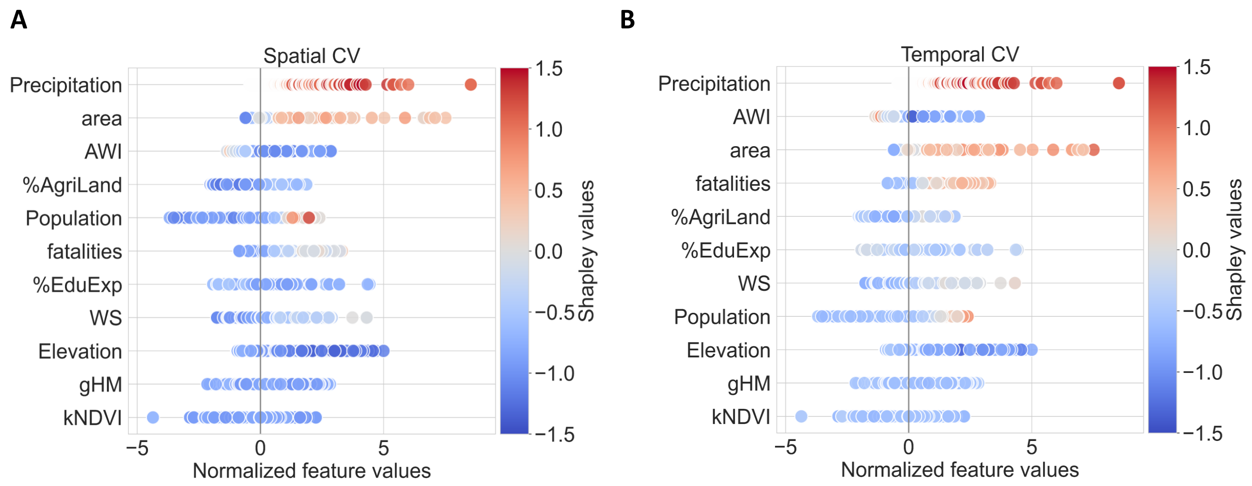

Figure 10. Shapley values. The horizontal axis represents the normalized feature values. At the same time, the color scale is determined by the mean Shapley values obtained with (A) spatial country-based splits or (B) temporal splits based on values of month and year.

the second most impacted continent. At the same time, South and North America are the least affected continents among the areas considered. In Fig. 12, we show the kernel density approximation of the distribution of the logarithm of NDP per event. The total number and average of NDP at different aggregation levels are summarised in Table 3, Table 4, and Table 5.

Table 3. Total number and average NDP per hazard type.

Hazard type	Sum NDP	Average NDP
Flooding	7,997,967	6,171
Landslide	139,626	509
Storm	7,818,646	9,420

Table 4. Total number and average NDP per continent.

Continent	Sum NDP	Average NDP
Africa	2,558,774	3,588
Asia	11,782,368	9,769
North America	1,202,206	7,561
South America	412,891	1,282

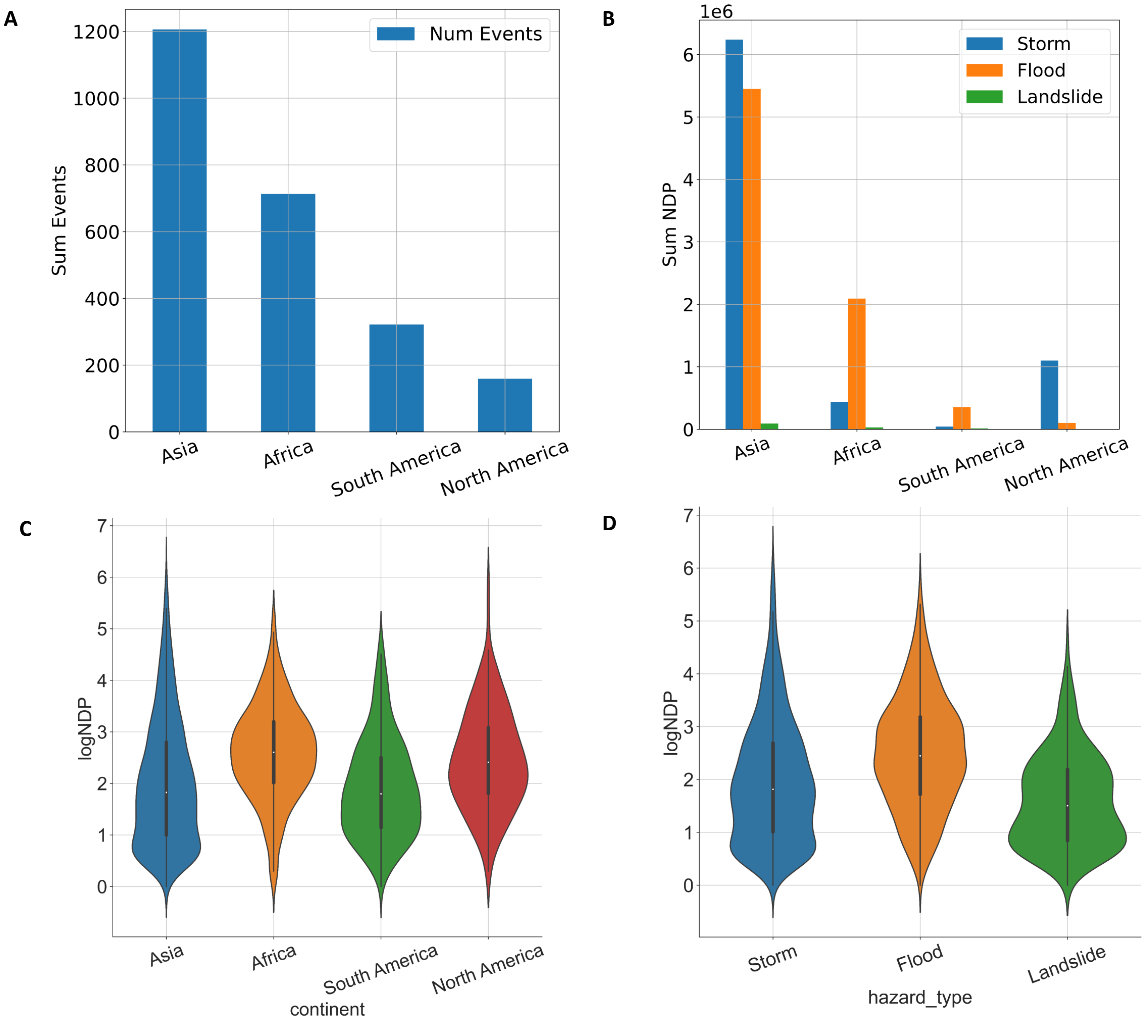

Figure 11. Distribution of NDP and events in the period 2016-2021 at different aggregation levels. (A) Histogram of the events per continent. (B) The total sum of NDP per continent disaggregated for the three types of hazards considered. (C) Violin plot of the (logarithm of) NDP per continent. (D) Violin plot of the (logarithm of) NDP per hazard type.

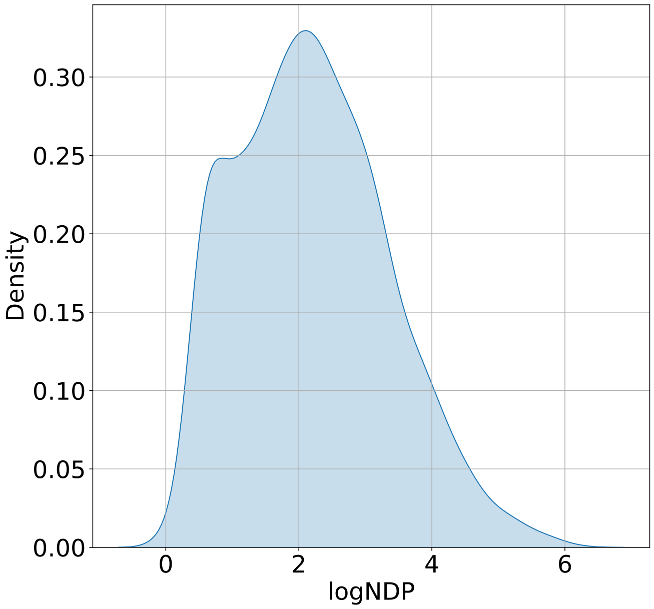

Figure 12. Density plot of the logarithm of NDP.

Table 5. Total number and average NDP per country.

Country	Sum NDP	Average NDP	Event Count
Algeria	3,444	431	8
Angola	55,217	2,045	27
Argentina	24,371	641	38
Bangladesh	146,505	39	37
Belize	6,273	2,091	3
Benin	44,582	7,430	6
Bhutan	120	60	2
Bolivia	3,774	377	10
Botswana	2,414	1,207	2
Brazil	134,968	3,067	44
Burundi	190,743	1,177	162
Cabo Verde	750	750	1
Cameroon	141,465	15,718	9
Central African Republic	37,280	1,243	30
Colombia	157,645	1,995	79
Comoros	19,372	19,372	1
Congo	169,897	42,474	4
Costa Rica	9,804	613	16
Côte d'Ivoire	5,618	561	10
Dem. Rep. Congo	605,752	9,615	63
Djibouti	9,376	4,688	2
Dominican Republic	73,632	4,602	16
Ecuador	2,368	118	20
El Salvador	19,032	1,359	14
Ethiopia	39,552	4,395	9

Table 5. Total number and average NDP per country.

Country	Sum NDP	Average NDP	Event Count
Gabon	2	2	1
Gambia	23,607	4,721	5
Ghana	88,968	2,471	36
Guatemala	7,309	1,827	4
Guinea	8,595	1,433	6
Guinea-Bissau	414	414	1
Guyana	168	56	3
Haiti	15,148	1,515	10
Honduras	954,853	53,047	18
India	5,597,183	45,139	124
Indonesia	423,981	1,710	248
Jamaica	25	8	3
Jordan	2,138	1,069	2
Kazakhstan	69	69	1
Kenya	84,769	3,260	26
Kyrgyzstan	4,806	1,202	4
Lao PDR	133,802	19,115	7
Lesotho	2,129	1,065	2
Liberia	19,408	2,773	7
Madagascar	8,170	1,362	6
Malawi	165,606	5,342	31
Malaysia	177,448	1,069	166
Maldives	20	20	1
Mali	3,187	637	5
Mauritania	6,630	6,630	1
Mauritius	4,680	669	7
Mexico	114,690	1,765	65
Mongolia	4,143	1,381	3
Mozambique	50,366	4,579	11
Namibia	203	68	3
Nepal	208,440	2,217	94
Nicaragua	1,440	144	10
Niger	9,123	9,123	1
Nigeria	128,877	2,864	45
Paraguay	73,484	18,371	4
Peru	16,113	130	124
Philippines	2,422,305	20,186	120
Rwanda	61,376	6,820	9
Senegal	8,907	2,227	4
Sierra Leone	5,318	5,318	1
South Africa	7,042	503	14
Sri Lanka	319,618	1,433	223
Tanzania	99,739	4,750	21
Uganda	381,653	3,635	105
Uzbekistan	70,000	70,000	1
Viet Nam	2,271,790	13,132	173
Zambia	8,625	784	11
Zimbabwe	5,5918	2,796	20

The predictors were chosen to characterize all three main axes of the problem: hazard, vulnerability, and exposure. A wide

variety of features were considered; among them, those with the most predictive power and better semantic meaning were selected. In particular, the hazard intensity is summarized by the maximum wind speed and maximum precipitation. Both features were extracted at a polygon level with GEE using the ERA5-Land dataset provided by the Copernicus Climate Change Service.

The exposure is contained in five main predictors, namely the area of the polygon, the mean kNDVI and the percentage of agricultural land (land exposure), the mean elevation (land exposure), and the population of the affected area (human exposure). Indeed, it is known that natural disasters affect mainly coastal and tropical areas. The kNDVI was computed as:

$$kNDVI = \tanh\left(NDVI^2\right) . \tag{5}$$

Finally, the vulnerability is characterized by AWI, the gHM, the percentage of education expenditures (concerning the gross national income), and conflict fatalities, which refers to the total number of deaths due to violent conflicts (i.e., human security threats and vulnerability). The AWI was computed as:

$$AWI_{ic} = rank_{ic} \times \frac{GDP_c}{\frac{1}{n}\sum_j ICDF_c(rank_{jc})}, \tag{6}$$

where $rank_{ic}$ is the rank of the pixel i of the country c as given by the RWI in c; GDP_c is the GDP per capita of the country c; and $ICDF_c$ is the inverse cumulative distribution function for a log-normal probability distribution.

In Fig. 14, we show the matrix of Pearson correlations among all the predictors and the target variable. Notice that most values are well below 50%, meaning that predictors are almost independent between them and potentially represent a good choice of variables. In Fig. 15, we plot the distributions and the box plots of all the features and also of the target variable.

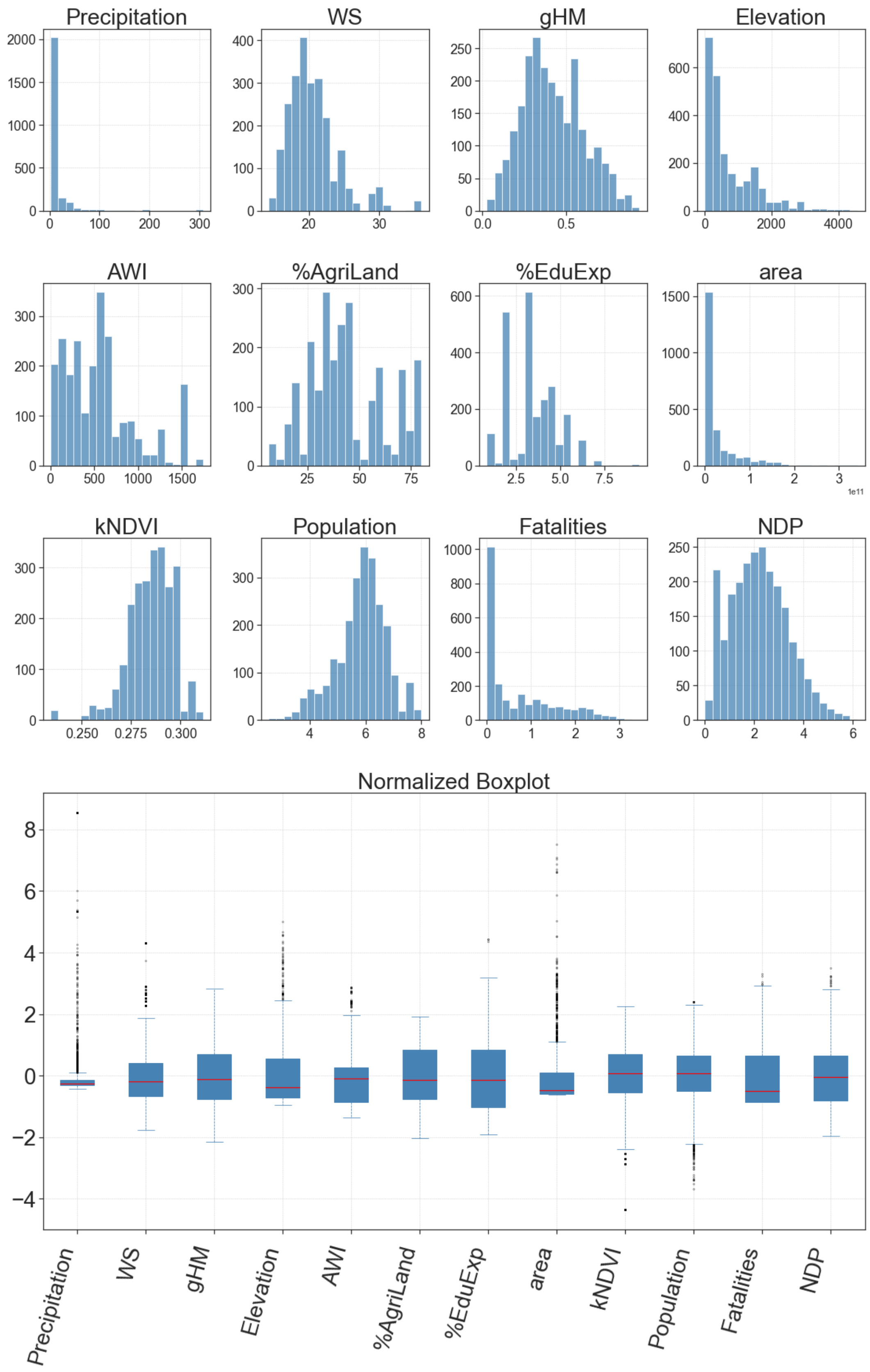

Figure 13. Histograms (up) and normalized boxplots (down) of the variables in the dataset.

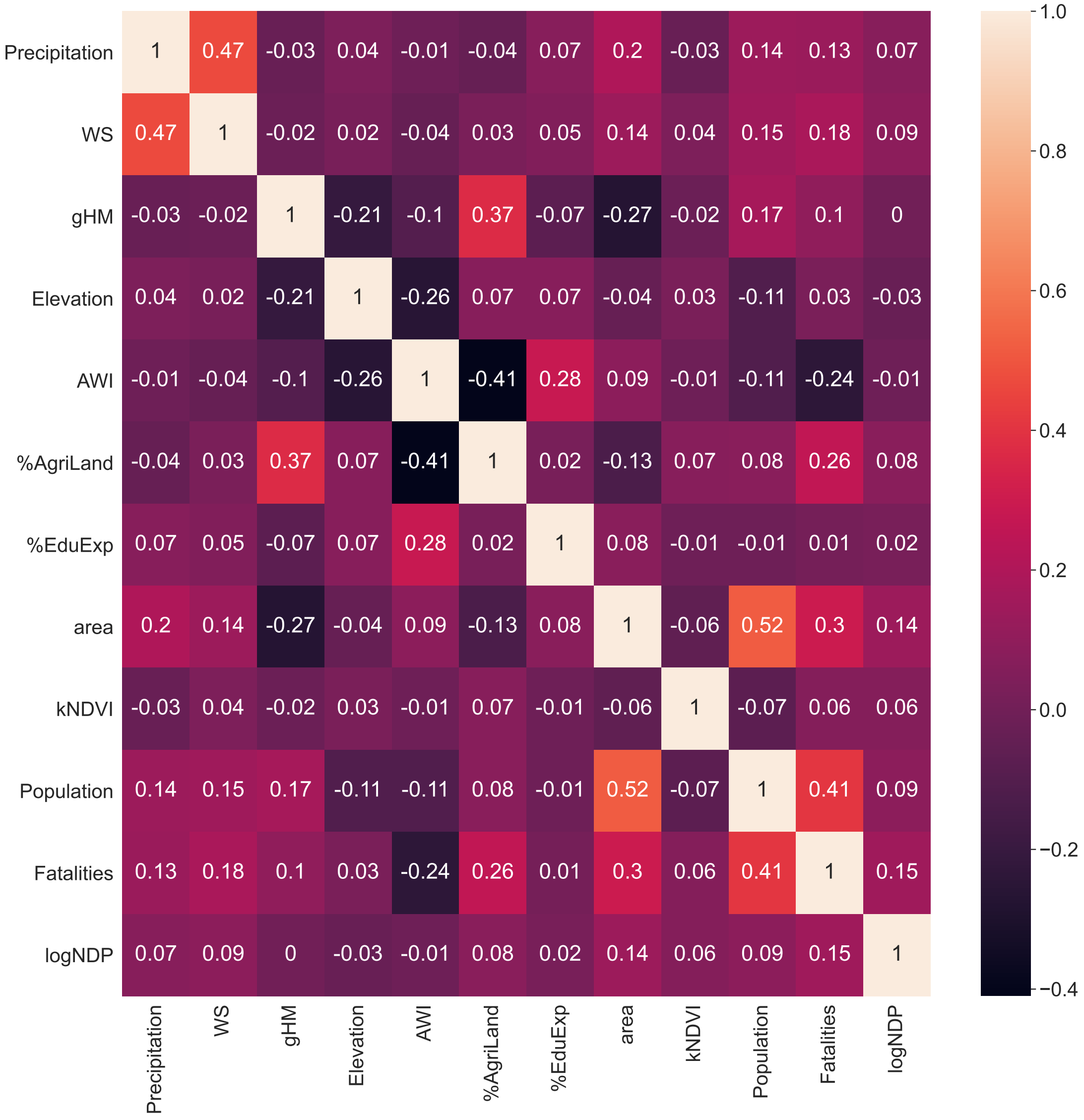

Figure 14. Pearson's correlation matrix between all covariates and with the target variable (logNDP).

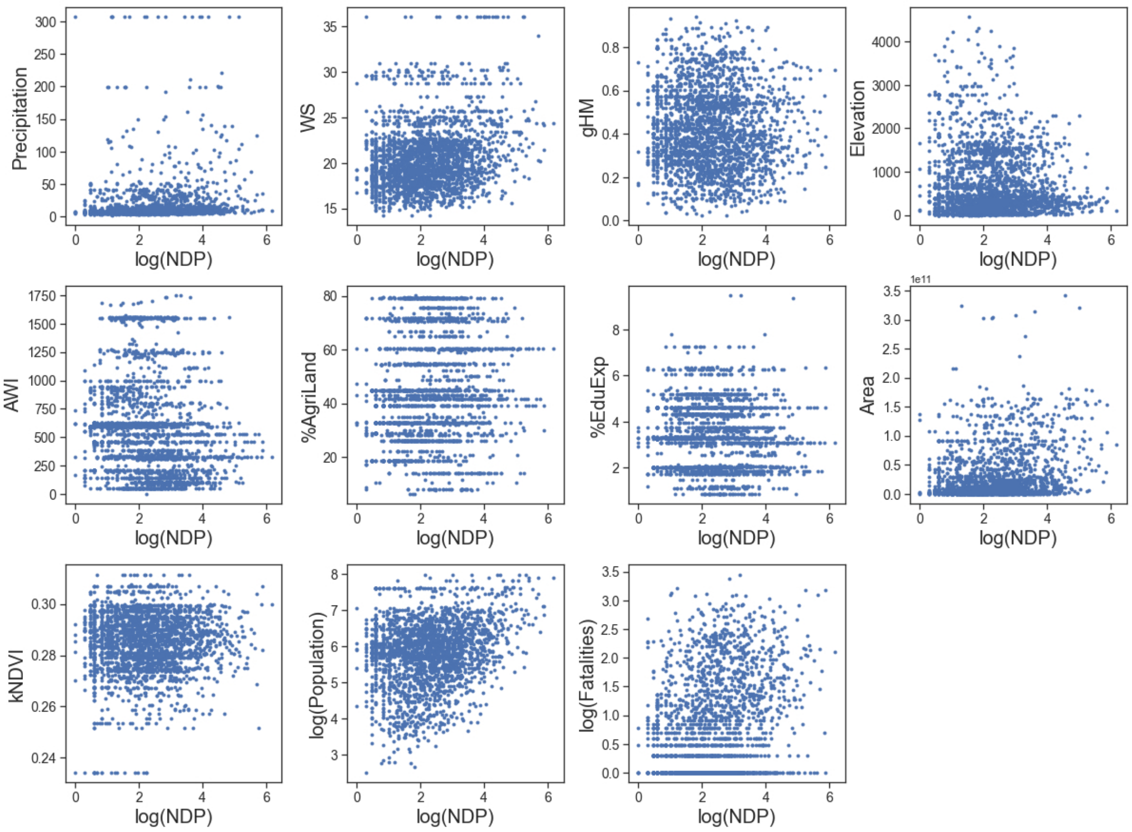

Figure 15. Scatter plots between log(NDP) and the covariates in the dataset.
