# Supplementary material for: Exploring interactions between socioeconomic context and natural hazards on human population displacement
Source: Nat Commun. 2023 Dec 4;14:8004. doi: 10.1038/s41467-023-43809-8 (PMC10695951; doi:10.1038/s41467-023-43809-8)
Supplement: Supplementary file 3 — Reporting Summary [file 41467_2023_43809_MOESM3_ESM.pdf]

Corresponding author(s): Michele Ronco

Last updated by author(s): Nov 1, 2023

Reporting Summary

Nature Portfolio wishes to improve the reproducibility of the work that we publish. This form provides structure for consistency and transparency in reporting. For further information on Nature Portfolio policies, see our Editorial Policies and the Editorial Policy Checklist.

Statistics

For all statistical analyses, confirm that the following items are present in the figure legend, table legend, main text, or Methods section.

n/a	Confirmed	
☐	☒	The exact sample size (*n*) for each experimental group/condition, given as a discrete number and unit of measurement
☐	☒	A statement on whether measurements were taken from distinct samples or whether the same sample was measured repeatedly
☒	☐	The statistical test(s) used AND whether they are one- or two-sided<br>*Only common tests should be described solely by name; describe more complex techniques in the Methods section.*
☐	☒	A description of all covariates tested
☐	☒	A description of any assumptions or corrections, such as tests of normality and adjustment for multiple comparisons
☐	☒	A full description of the statistical parameters including central tendency (e.g. means) or other basic estimates (e.g. regression coefficient) AND variation (e.g. standard deviation) or associated estimates of uncertainty (e.g. confidence intervals)
☒	☐	For null hypothesis testing, the test statistic (e.g. *F*, *t*, *r*) with confidence intervals, effect sizes, degrees of freedom and *P* value noted<br>*Give P values as exact values whenever suitable.*
☒	☐	For Bayesian analysis, information on the choice of priors and Markov chain Monte Carlo settings
☒	☐	For hierarchical and complex designs, identification of the appropriate level for tests and full reporting of outcomes
☐	☒	Estimates of effect sizes (e.g. Cohen's *d*, Pearson's *r*), indicating how they were calculated

Our web collection on statistics for biologists contains articles on many of the points above.

Software and code

Policy information about availability of computer code

Data collection

Data were collected using four primary methods:

1. Direct Download: Data was obtained by directly downloading from the sources and web links provided, with further details on data availability.
2. Google Earth Engine (GEE): Google Earth Engine was used as a method for data collection.
3. Data Sharing: Data providers shared information in the form of .csv or .xlsx files.
4. Open APIs: Open and free APIs were used to retrieve specific data.

The following variables were collected using the mentioned methods:
Method 1: AWI, ACLED, and Area.
Method 2: Precipitation, wind speed, population, elevation, gHM, and kNDVI.
Method 3: New Displacements of People (NDPs).
Method 4: Education expenditures and fraction of agricultural land.

As explained in the Methods section ("Building a Global Dataset of Displacements"), displacements recorded by the IDMC contain location names which were converted into shapefiles using (a python package based on) Open Street Map. These shapefiles were uploaded on GEE and used to aggregate some of the covariates (those collected using method 4). The function used for the aggregation are listed in Table 2.

Data analysis

Data analysis was conducted using custom Python code tailored for the specific analytical tasks. The dataset and the code, along with its dependencies, are available on Zenodo at the link: https://zenodo.org/records/10063853 .

In particular, we used Python 3.7.10, with the following main libraries:
- OSMnx installed from https://github.com/gboeing/osmnx , for extracting polygon shapefiles from hazard location names.
- Pandas 1.3.0 for data preprocessing and harmonization
- Matplotlib 3.4.2 and Seaborn 0.11.1 for creating plots and figures
- Scikit-learn 0.24.2 for statistical analysis and machine learning modeling
- Shapley package 1.7.1 for explainable AI analysis

In addition to these main libraries, various other packages and their dependencies were employed in the analysis. All utilized packages, including their dependencies, are open-source and can be readily installed using pip or conda.

For manuscripts utilizing custom algorithms or software that are central to the research but not yet described in published literature, software must be made available to editors and reviewers. We strongly encourage code deposition in a community repository (e.g. GitHub). See the Nature Portfolio guidelines for submitting code & software for further information.

Data

Policy information about availability of data

All manuscripts must include a data availability statement. This statement should provide the following information, where applicable:

- Accession codes, unique identifiers, or web links for publicly available datasets
- A description of any restrictions on data availability
- For clinical datasets or third party data, please ensure that the statement adheres to our policy

All data used for this study came from open source databases. In particular, the following variables were collected and harmonized:
- New displacements of people (NDP) from the Internal Displacement Monitoring Centre (IDMC). NDP can be downloaded from the IDMC data portal at: https://www.internal-displacement.org/database/displacement-data .
- Absolute Wealth Index (AWI) was derived from the RWI downloaded from the Meta Data4Good portal at: https://dataforgood.facebook.com/dfg/tools/relative-wealth-index .
- Maximum precipitation values were retrieved from Google Earth Engine: https://developers.google.com/earth-engine/datasets/catalog/ECMWF_ERA5_LAND_HOURLY . The original source is the ERA5 Land dataset from ECMWF which can be downloaded also from the Copernicus Climate Data Store : https://cds.climate.copernicus.eu/cdsapp#!/dataset/reanalysis-era5-land?tab=overview .
- Maximum wind speed values were retrieved from Google Earth Engine: https://developers.google.com/earth-engine/datasets/catalog/ECMWF_ERA5_LAND_HOURLY . The original source is the ERA5 Land dataset from ECMWF which can be downloaded also from the Copernicus Climate Data Store : https://cds.climate.copernicus.eu/cdsapp#!/dataset/reanalysis-era5-land?tab=overview .
- kNDVI was derived from the NDVI from Google Earth Engine: https://developers.google.com/earth-engine/datasets/catalog/MODIS_MOD09GA_006_NDVI . The original source of the Vegetation Indices 16-Day L3 Global 500m is the MODIS Terra MOD13A1 dataset at: https://modis.gsfc.nasa.gov/data/dataprod/mod13.php . Tools other than GEE are also available.
- Gridded population values were obtained with Google Earth Engine: https://developers.google.com/earth-engine/datasets/catalog/CIESIN_GPWv411_GPW_Population_Count . The original data source is the SEDAC NASA Data Center: https://sedac.ciesin.columbia.edu/data/set/gpw-v4-population-count-rev11 , which also provide direct data download.
- Global Human Modification map was obtained from Google Earth Engine: https://developers.google.com/earth-engine/datasets/catalog/CSP_HM_GlobalHumanModification . This data layer is produced by Conservation Science Partners the https://csp-inc.org .
- Elevation data was taken from the SRTM 90m Digital Elevation Model at: https://srtm.csi.cgiar.org .
- People fatalities produced by conflicts were taken from the ACLED website at: https://acleddata.com/data-export-tool/ .
- The areas of the polygons of the locations affected by storms, floods, and landslides were computed from Open Street Map: https://nominatim.openstreetmap.org/ui/search.html .
- Education expenditures were obtained from the SDG API at: https://unstats.un.org/SDGAPI/swagger/ , provided by the United Nations Statistics Department: https://unstats.un.org/UNSDWebsite/ .
- Fraction of Agricultural Land at the country level was retrieved from the SDG API at: https://unstats.un.org/SDGAPI/swagger/ , provided by the provided by the United Nations Statistics Department: https://unstats.un.org/UNSDWebsite/ .

The harmonized dataset we used for our study is freely available on Zenodo at: https://zenodo.org/records/10063853 .

Research involving human participants, their data, or biological material

Policy information about studies with human participants or human data. See also policy information about sex, gender (identity/presentation), and sexual orientation and race, ethnicity and racism.

Reporting on sex and gender	No sex or gender information was used or reported in our study.
Reporting on race, ethnicity, or other socially relevant groupings	No race or ethnicity information was used or reported in our study.
Population characteristics	We selected records from the full IDMC dataset of new displacements that belong to middle and low-income countries, as defined by the availability of the Absolute Wealth Index (AWI), as specified in our study.
Recruitment	We did not use participants in our study.
Ethics oversight	Internal Displacement Monitoring Centre (IDMC), Universidad de Valencia (UV), Image Processing Laboratory (IPL)

Note that full information on the approval of the study protocol must also be provided in the manuscript.

Field-specific reporting

Please select the one below that is the best fit for your research. If you are not sure, read the appropriate sections before making your selection.

☐ Life sciences ☒ Behavioural & social sciences ☐ Ecological, evolutionary & environmental sciences

For a reference copy of the document with all sections, see nature.com/documents/nr-reporting-summary-flat.pdf

Behavioural & social sciences study design

All studies must disclose on these points even when the disclosure is negative.

Study description	The primary objective of this study was to analyze the factors driving human displacements triggered by sudden-onset weather hazards such as storms, floods, and landslides. Specifically, our research focused on investigating the interplay among climate conditions, economic factors, and exposure variables to gain insights into the mechanisms that render communities most susceptible to natural disasters. To achieve this goal, we constructed and harmonized a comprehensive global dataset of New Displacements of People (NDPs) per disaster for the years 2016-2021. For each event, we obtained the geographical extent of the affected area in the form of geolocalized polygons. These polygons served as the basis for aggregating weather, exposure, and socioeconomic data. All data used in the study were quantitative, and the units of measurement were provided in the text. Variables were rescaled for modeling to facilitate comparisons among diverse factors. Machine learning techniques, specifically Random Forest and Gradient Boosting models, were employed with cross-validation to predict NDPs for each weather-related event. To interpret the predictions and unearth the key drivers of human displacements, Shapley values were computed and thoroughly analyzed. This allowed us to gain a deeper understanding of the underlying mechanisms shaping human responses to natural disasters in terms of hazard intensity, economic vulnerability and people exposure.
Research sample	The sample of interest for the study included displaced persons in low and middle income countries which are believed to be the most vulnerable to weather hazards according both to experts and to previous studies appeared in the literature . The selection was performed by relying on the AWI which is a recently introduced index quantifying micro-scale households poverty conditions. To our knowledge, this is the most accurate sample on people displaced due to sudden-onset disasters in middle and low income countries.
Sampling strategy	In the data collection phase, no specific sampling strategy was employed. However, in the modeling phase, we implemented a distinct sampling strategy along with randomization. We adopted a stratified sampling approach, which involved discretizing (or binning) the values of the target variable, specifically, the count of new displaced people (NDPs). We divided the data into 10 discrete intervals using quantiles to ensure that the histogram of the binned values closely mirrored the original distribution of continuous NDP data. To train Machine Learning models, we then randomly divided the dataset into training and testing sets while maintaining the stratification of the target variable. This approach ensured that both the training and testing sets had a distribution of NDPs that closely resembled that of the entire dataset, enhancing the model's performance.
Data collection	The predictor variables were collected using Google Earth Engine, open source APIs, and direct data downloads from web links provided.<br><br>The target variable, which represents the number of newly displaced people, was sourced from IDMC. IDMC is a global authority on monitoring people's displacements, particularly those resulting from natural disasters and conflicts. Details about IDMC's data collection process can be found at: https://www.internal-displacement.org/monitoring-tools . IDMC's data collection approach involves tracking individual displacement events, capturing location, date, triggers, causes, and duration. They collect data from a range of sources with different priorities, including governments, international organizations, NGOs, and media reports, which are triangulated for the most accurate displacement estimates. Their data collection methods, definitions, and standards are consistent across countries and time. IDMC records all displacement events based on data availability, without specific thresholds, covering both cumulative movements over time (flows) and static counts at specific points in time (stocks). For this study, we relied solely on flow data. Additionally, IDMC recognizes the variety of terms and scenarios used to describe internal displacement by different sources, including forced movements, evacuations, relocations, sheltered populations, homelessness, and housing conditions. This comprehensive approach ensures accurate monitoring and interpretation of displacement data. As of now, IDMC's data is considered the most accurate source for tracking displacements triggered by disasters.
Timing	We used data from 04/2016 until 12/2021. This choice was mainly motivated by the fact that IDMC data earlier than 2016 can contain more gaps.
Data exclusions	The selection criterion, based on the Absolute Wealth Index (AWI), was consistently applied as it serves as the primary indicator of local economic vulnerability within the modeling process. Preliminary analyses of the entire dataset were deliberately avoided to ensure an unbiased selection and to mitigate potential biases.
Non-participation	No participants were involved in the study.
Randomization	Various randomization schemes were employed for model training and validation. Bootstrapping was used to assess model performance, with each bootstrap iteration randomly splitting the data into 30% for testing and 70% for training. The initial stratified sampling procedure used 10 discrete levels for the target variable. For spatial cross-validation, we divided the data by the country of the event, ensuring different countries were randomly assigned to either the training or test sets in each bootstrap iteration. In temporal cross-validation, a similar procedure was followed, but the division was based on the event's year and month, rather than the country.

Reporting for specific materials, systems and methods

We require information from authors about some types of materials, experimental systems and methods used in many studies. Here, indicate whether each material, system or method listed is relevant to your study. If you are not sure if a list item applies to your research, read the appropriate section before selecting a response.

Materials & experimental systems

n/a	Involved in the study
☒	☐ Antibodies
☒	☐ Eukaryotic cell lines
☒	☐ Palaeontology and archaeology
☒	☐ Animals and other organisms
☒	☐ Clinical data
☒	☐ Dual use research of concern
☒	☐ Plants

Methods

n/a	Involved in the study
☒	☐ ChIP-seq
☒	☐ Flow cytometry
☒	☐ MRI-based neuroimaging

Plants

Seed stocks	NA
Novel plant genotypes	NA
Authentication	NA
